# Connective tissue growth factor dependent collagen gene expression induced by MAS agonist AR234960 in human cardiac fibroblasts

**DOI:** 10.1371/journal.pone.0190217

**Published:** 2017-12-29

**Authors:** Arunachal Chatterjee, John Barnard, Christine Moravec, Russell Desnoyer, Kalyan Tirupula, Sadashiva S. Karnik

**Affiliations:** 1 Department of Molecular Cardiology, Lerner Research Institute, Cleveland Clinic, Cleveland, Ohio, United States of America; 2 Department of Zoology, Acharya Jagadish Chandra Bose College, Kolkata, West Bengal, India; 3 Department of Quantitative Health Sciences, Lerner Research Institute, Cleveland Clinic, Cleveland, Ohio, United States of America; 4 Department of Cardiovascular Medicine, Heart and Vascular Institute, Lerner Research Institute, Cleveland Clinic, Cleveland, Ohio, United States of America; University of North Dakota, UNITED STATES

## Abstract

Perspectives on whether the functions of MAS, a G protein-coupled receptor, are beneficial or deleterious in the heart remain controversial. MAS gene knockout reduces coronary vasodilatation leading to ischemic injury. G protein signaling activated by MAS has been implicated in progression of adaptive cardiac hypertrophy to heart failure and fibrosis. In the present study, we observed increased expression of MAS, connective tissue growth factor (CTGF) and collagen genes in failing (HF) human heart samples when compared to non-failing (NF). Expression levels of MAS are correlated with CTGF in HF and NF leading to our hypothesis that MAS controls CTGF production and the ensuing expression of collagen genes. In support of this hypothesis we show that the non-peptide MAS agonist AR234960 increases both mRNA and protein levels of CTGF via ERK1/2 signaling in HEK293-MAS cells and adult human cardiac fibroblasts. MAS-mediated CTGF expression can be specifically blocked by MAS inverse agonist AR244555 and also by MEK1 inhibition. Expression of CTGF gene was essential for MAS-mediated up-regulation of different collagen subtype genes in HEK293-MAS cells and human cardiac fibroblasts. Knockdown of CTGF by RNAi disrupted collagen gene regulation by the MAS-agonist. Our data indicate that CTGF mediates the profibrotic effects of MAS in cardiac fibroblasts. Blocking MAS-CTGF-collagen pathway should be considered for pharmacological intervention for HF.

## Introduction

Heart failure (HF) in humans, characterized by a heart unable to pump blood to meet the body’s needs, is one of the most common causes of mortality [1]. In acquired and genetic forms of HF, left ventricular hypertrophy (LVH) combined with reduced ejection fraction and progressive interstitial collagen accumulation account for the ventricular dysfunction. Activation of the local renin-angiotensin-aldosterone system (RAAS) is thought to be a determinant of myocardial failure independent of cardiac hypertrophy [2,3]. RAAS regulates the production of hormone peptides (angiotensin II (AngII), angiotensin 1–7 (Ang1-7)) and aldosterone as an adaptive response to pathophysiological stress. Inhibiting the actions of AngII and aldosterone demonstrated beneficial preservation of cardiac functions after myocardial ischemia and diabetes [4,5]. Additional RAAS blockade approaches that regress excess collagen production in LVH need to be developed to determine if myocardial fibrosis is indeed reversible in humans.

Connective tissue growth factor (CTGF) is recognized as a critical regulator of myocardial extracellular matrix, including changes in the composition of collagen, an interstitial fibrosis marker [6]. CTGF is a downstream autocrine/paracrine factor regulated by hormones including AngII, TGFβ, growth factors and cytokines. CTGF regulates fibroblast proliferation, collagen synthesis and apoptosis, processes which accelerate cardiac fibrosis in pathological conditions such as diabetes or ischemic heart disease [6]. CTGF promoted myofibroblast differentiation [7] may underlie multiple diseases including renal [8] lung [9] and cardiac fibrosis in diabetes [10]. Although the role of CTGF in developmental biology and cancer has been widely studied, its regulation in cardiac biology is not well characterized.

Regulatory mechanisms connecting different RAAS hormones to CTGF production may be involved in myocardial fibrosis [4,11]. Three different GPCRs, AT_1_R, AT_2_R and MAS mediate RAAS effects on cells [4,12]. Of these AngII-activation of AT_1_R has been shown to increase collagen production in cardiac fibroblasts and AngII-activation of AT_2_R may inhibit this process [4]. Infusion of AngII was shown to increase CTGF mRNA, which was inhibited by the AT_1_R antagonist candesartan [13]. However, the role of MAS in regulating CTGF and collagen production in cardiac tissue is unknown. The cardiac effects of MAS gene deletion vary in different studies. Castro et al. have shown that MAS agonist Ang1-7 improves coronary blood flow and this effect is absent in MAS gene deleted mice [14,15]. In contrast, long-term cardioprotection after ischemia was shown by inhibition of MAS leading to improved coronary blood flow [16]. A review of signaling studies reveal that MAS activates calcium signaling similar to AT_1_R, hence it may promote cardiac fibrosis [12, 16–18]. Further, MAS has been shown to act as a critical regulator of renal fibrogenesis and produce pro-inflammatory effects as potent as those produced by AT_1_R and TNFαR [19, 20]. Thus, MAS may alter cardiac tissue composition in addition to regulating coronary flow. However, MAS regulated gene expression changes are not well understood, and surprisingly little progress has been made in determining the relation between CTGF, collagens and MAS.

Our aim was to investigate fibrogenic actions of MAS involving CTGF and collagen genes. MAS was discovered as a growth promoting GPCR that increased intracellular calcium [4,12,21,22,] and was later shown to produce arachidonic acid in MAS-transfected cells and vasodilatation in response to Ang1–7 [12, 23]. A review of recent literature revealed that Ang1–7 activates at least three GPCRs including MAS, AT_2_R and MAS-related receptor, MRGD [4, 12]. To selectively activate MAS in this study, we used the MAS-specific non-peptide agonist AR234960 [16,18] and demonstrated that CTGF expression is activated which in turn regulates collagen expression. Both effects are blocked by the MAS-specific inverse agonist AR244555.

## Materials and methods

### Human cardiac tissue samples

Human heart samples, non-failing (NF) and failing (HF) were obtained from explanted hearts of transplant recipients at department of Cardiovascular Medicine at Cleveland Clinic (Cleveland, OH). Only left ventricular (LV) free wall tissue samples were included in this study. The NF control heart tissues with normal ventricular structure and function were obtained from unmatched donors whose hearts were not suitable for transplantation. Written informed consent from the donor or the next of kin was obtained for the use of these samples in research under Cleveland Clinic Institutional Review Board protocol #2378, which specifically approved this study. The investigation also conforms to the principles outlined in the *Declaration of Helsinki*” (*Br Med J* 1964; ii: 177). Sample size was 10 per each group for NF and 3 different HF groups based on clinical description described in Table 1.

**Table 1:** Patient demographics. The abbreviations used for non-failing hearts are as follows: F, female; M, male; W, white; EF, left ventricular ejection fraction measured prior to explant; CVA, cerebrovascular accident; GSW, gunshot wound. Drug therapy acute indicates treatment in the emergency room or intensive care unit prior to brain death: Drugs: DOB, dobutamine (n = 2); DOP, dopamine (n = 4); EPI, epinephrine (n = 1); NE, norepinephrine (n = 4); THY, thyroxine (n = 4), other, nimodipine, phenylephrine, vasopressin, atropine (n = 1 or 3). Drug therapy chronic, indicates drugs (phenobarbital, coumadin, spironolactone, synthroid) taken by patients prior to admission, as reported by family members (n = 1). The abbreviations used for failing hearts are as follows: F, female; M, male; W, white; B, black; NatAm, Native American; A, asian; O, other; EF, left ventricular ejection fraction measured prior to explant; DCM, dilated cardiomyopathy (pre-transplant diagnosis); ICM, ischemic cardiomyopathy; VCM, valvular cardiomyopathy. Drug therapy lists as follows: AM, amiodarone (n = 13); CAPT, captopril (n = 4); CARV, carvedilol (n = 8); DIG, digoxin (n = 10); DOB, dobutamine (n = 6); EN, enalapril (n = 1); LIS, lisinopril (n = 7); MET, metoprolol (n = 7); MIL, milrinone (n = 10); dofetilide, losartan, mexiletine, fosinopril, trazodone, colchicine (n = 1 or 2).

	Age	Sex	Race	EF%	Cause of death	Drug therapy
**Non-failing hearts** **(*n = 1*0)**	48.5±4.6	4F, 6M	10W	61.0±2.11	7 CVA, 2 GSW, 1 Anoxia	Acute: DOB, DOP, EPI, NE, THY, other medicines; Chronic medicines
**Failing hearts** **(*n = 3*0)**	57.6±2.0	9F, 21M	25W, 2B, 1NatAm, 1A, 1O	21.2±2.55	10 DCM, 10 ICM, 10 VCM	AM, CAPT, CARV, DIG, DOB, EN, LIS, MET, MIL, dofetilide, losartan, mexiletine, fosinopril, trazodone, colchicine

### Cell cultures and treatment

Tetracycline/doxycycline inducible T-Rex HEK293 cell lines stably transfected with N-terminal myc-tagged MAS (HEK293-MAS) were used. MAS expression was induced in stable cells with doxycycline (100ng/ml) for 12h [18]. After induction cells were treated either with MAS-specific non-peptide agonist (AR234960; Ago; 10μM) or in combination with MAS-specific non-peptide inverse-agonist (AR244555; Inv; 10μM) from Arena Pharmaceuticals (San Diego, CA) for 12 h. Induced cells without ligand treatment were used as controls in the experiments.

Adult primary human cardiac fibroblasts (HCF; 306K-05f; Cell Applications, CA) were grown and maintained in Lung/Cardiac Fibroblast Growth Media (Cell Applications, CA) with 10% FBS and Penicillin/Streptomycin (Invitrogen, NY). During the experiment HCF cells were treated with MAS-specific Ago (10μM) or in combination with MAS-specific Inv (10μM). MEK1 activity was blocked by pretreating the cells with 10μM MEK1 inhibitor (iMEK1; PD98059; Cell Signaling, MA) for 2h before adding MAS-specific agonist. To silence the CTGF expression in HCF and HEK293-MAS cells, 5nM Control siRNA (Cat#4390843; Ambion) and CTGF siRNA (s3708; Cat#4390824; Ambion) was transiently transfected using RNAiMAX (Invitrogen) following manufacturer’s protocol for 20 h, followed by treatment with MAS agonist for 12h.

### Real time quantitative-PCR (RT-qPCR)

Total RNA was isolated from human left ventricular heart tissues and cells using miRNeasy Mini Kit (Qiagen, CA). Reverse transcription and qPCR were done using iScript Reverse Transcription Supermix and iQ-SYBR Green Supermix respectively (Bio-Rad, CA) using MyiQ2 Two Color Real Time PCR Detection System (Bio-Rad, CA) following manufacturer’s protocol. Expression of MAS, CTGF, and different sub-types of collagen genes were studied using forward and reverse primers (Invitrogen, NY) (Table 2). GAPDH gene was amplified as an internal control. Delta *C*(*t*) values for expression levels of genes were calculated relative to GAPDH gene expression, and the ΔΔCT method was used to compare expression among samples yielding log_2_-based expression values, which were converted to linear values by calculating 2^−ΔΔCT^. To calculate gene expression in human heart samples, relative log_2_ gene expression levels were fit to an additive linear model using the R statistical program. A multidimensional scaling was used as implemented in the R package by a professional bio statistician (www.lerner.ccf.org/qhs/genetics/). All correlation analyses were performed using Prism software (GraphPad).

**Table 2:** Primers used.

Gene (Human)	Forward primer	Reverse primer
CTGF	5′ GCAGGCTAGAGAAGCAGAGC 3′	5′ GGTGCAGCCAGAAAGCTC 3′
MAS	5′ GCTACAACACGGGCCTCTATCTG 3′	5′ TACTCCATGGTGGTCACCAAGC 3′
COL1A1	5′ AGCGTGGCCTACATGGAC 3′	5′ CGACAGTGACGCTGTAGGTG 3′
COL1A2	5′ CTCGCTCAGCACCTTCTCTC 3′	5′ CACTCTGGGTGGCTGAGTC 3′
COL3A1	5′ CTTCTCTCCAGCCGAGCTTC 3′	5′ GACCCCATCAGCTTCAGG 3′
COL4A1	5′ GTGTGCTGTGTGTGAGGC 3′	5′ TAGCCGATCCACAGCGAG 3′
COL4A2	5′ CGGGCAGCTGTCTAGAGG 3′	5′ TCTGCTCGGGAATGGTGG 3′
GAPDH	5′ AGCCAAAAGGGTCATCATCTCTG 3′	5′ CATGAGTCCTTCCACGATACCAAA 3′

### Western blot

Protein extracts were prepared from cells and human left ventricular tissue using M-PER and T-PER lysis buffer (Thermo Scientific, NY) respectively [24]. Immunoblotting was performed using primary antibody specific for CTGF (Santa Cruz, CA), phospho-ERK1/2, Total-ERK1/2 (Cell Signaling, MA) and GAPDH (Ambion, IL) followed by incubation with infrared dye (IRDye)-conjugated secondary antibodies (LI-COR, NE) and immunoreactive bands were visualized under Odyssey scanner (LI-COR, NE). The bands were quantitated using Image Studio Ver3.1 (LI-COR, NE) and normalized by GAPDH.

### Immunohistochemistry

Myocardial tissue cryo-sections (4μm) were stained with Masson’s trichrome stain to look for collagen and with antibody against CTGF. The same set of sections were stained for hematoxylin and eosin to check quality of the sections. Sections were analysed by bright field microscopy [2].

### Statistical analysis

All data were expressed either as mean ± SD or mean ± SEM (n≥3 experiments performed under identical conditions). Statistical analyses were performed using GraphPad Prism with an unpaired t test with Welch’s correction. p<0.05 were considered statistically significant.

## Results

### Expression of MAS is correlated with CTGF and fibrosis in human heart samples

We compared steady-state expression of MAS transcripts in the frozen human heart tissue LV wall, categorized as failing (HF) and non-failing (NF) samples. Detailed phenotypic parameters are summarized in Table 1 demonstrated that HF hearts had left ventricular ejection fraction (EF) that is significantly reduced in comparison to the NF group (S1 Fig). Mean age, 57.6±2.0 years for the HF donors was not significantly different from 48.5±4.6 years for NF donors. The NF donors had relatively normal ventricular function with no associated cardiac dysfunction as measured by echocardiography. Most donors were receiving some form of inotropic and/or vasopressor support. Total RNA was extracted from 10NF and 30HF donor samples and the quality assessment indicated that isolated RNA was intact.

**Fig 1:**
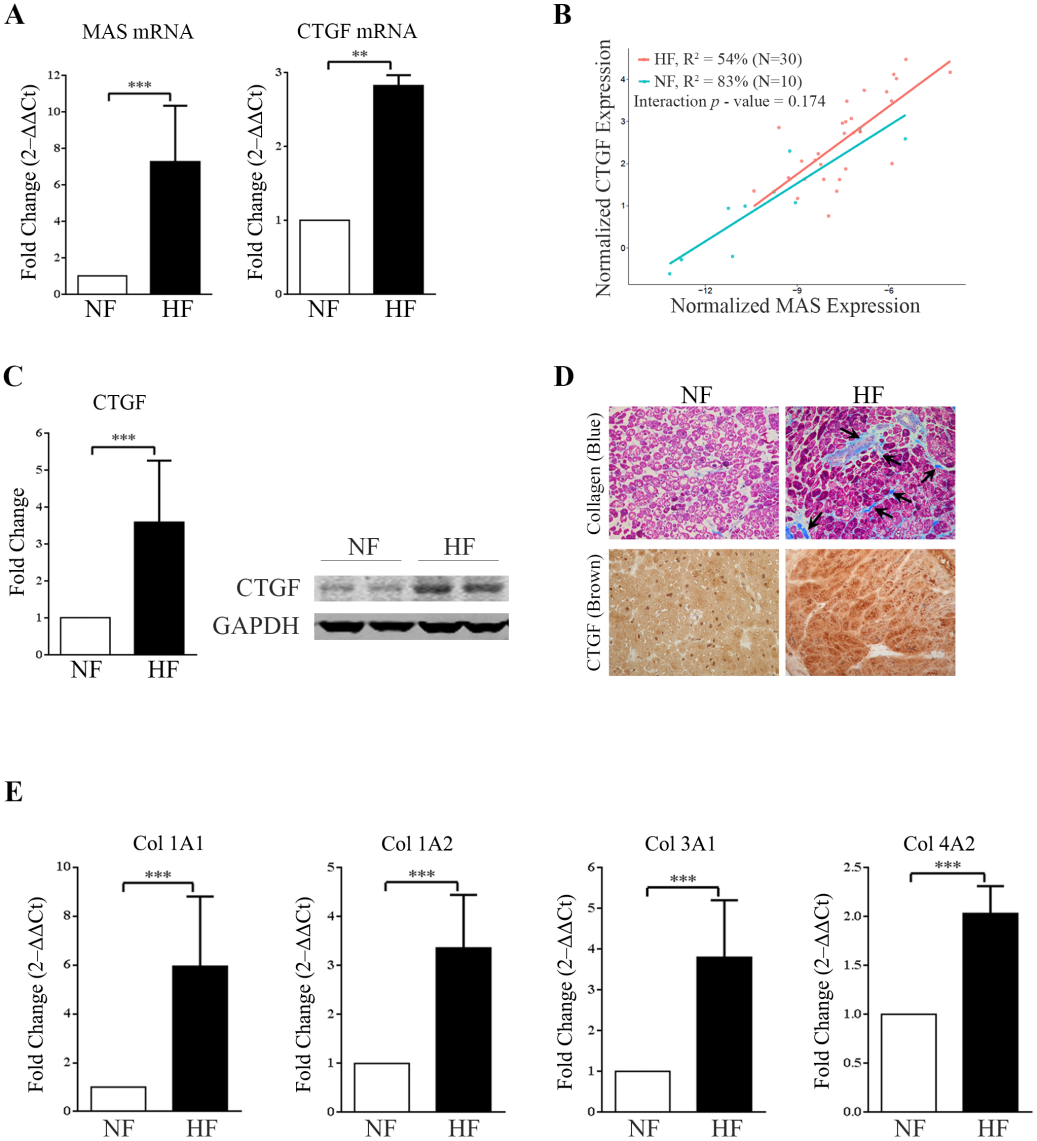
Upregulation of CTGF, MAS and collagen in human heart failure. **(A)** Bar graphs showing real-time PCR analysis of fold increase (2^‒ΔΔCt^) of MAS receptor mRNA (left) and CTGF mRNA (right) expression in tissue from failed human heart (HF) compared with non-failing (NF) samples (**p<0.01; ***p<0.001). Expression was normalized to GAPDH. **(B)** Correlation plot between normalized expression of CTGF and MAS mRNA showing strong interaction between them (*p* value–0.174). **(C)** Western-blot showing significant upregulation of CTGF in HF tissue samples compared to NF samples (right); GAPDH was used as loading control. The western blot image shown is representative of all the experiments done under similar experimental conditions and the data from multiple experiments quantitated and cumulative data were presented as bar graphs (left) (***p<0.001). **(D)** Masson’s Trichrome staining of cryo-sections (4μm) of human heart left ventricular wall tissue showing increased collagen deposition (stained blue) in inter-cellular spaces (arrows) of the HF samples compared to NF samples (upper panels); magnification ×20. Immuno-histochemical staining of same set of tissue sections with CTGF antibody, showing more deposition of CTGF (intense brown) in HF sections then NF samples (lower panels); magnification ×20. **(E)** Bar graphs showing real-time PCR analysis of different sub-types of collagen expression (Col 1A1, Col 1A2, Col 3A1 and Col 4A2) [represented as fold increase (2^‒ΔΔCt^)] in left ventricular heart tissue from failing (HF) as well as non-failing (NF) samples (***p<0.001). Expression was normalized to GAPDH. All the bar graphs are presented with error bar of ±SD.

RT-qPCR combined with statistical analyses demonstrated that MAS expression was significantly up regulated in the HF compared to NF. The CTGF mRNA expression in the HF samples was also markedly increased (>2.5 fold) compared to NF (Fig 1A). We observed high correlation between CTGF and MAS expression in both groups (Fig 1B); while similar correlation was not observed with expression levels of both type 1 and type 2 AngII receptors in these samples. Western blot analysis revealed increased CTGF protein level (Fig 1C). We also performed Masson’s trichrome staining of LV heart tissue cryo-sections to measure collagen, the marker for cardiac fibrosis. Significant staining of collagen deposition was observed in the intercellular spaces of HF compared to NF (Fig 1D). Immunostaining analysis also demonstrated increased CTGF expression in HF samples (Fig 1D). We examined the level of expression of different types of collagen transcripts (Col1A1, Col1A2, Col3A1, and Col4A2) in HF and NF heart samples. Expression of all collagen genes was significantly increased in HF group compared to NF group (Fig 1E). These findings led us to postulate that MAS regulated CTGF expression may contribute to up-regulation of collagen in HF human heart samples. In experimental rat models, AngII-induction of CTGF mRNA expression was found to lead to cardiac fibroblast activation during chronic HF [25]. Studies in feline isolated adult cardiomyocytes indicate that PE and AngII induced CTGF expression and release during maladaptive remodeling [26]. However, genetic overexpression of CTGF in rat cardiomyocytes was shown to promote cardiac hypertrophy but not fibrosis and protect against pressure overload [27]. Therefore, we proceeded to examine whether ligand modulation of MAS regulates CTGF expression in cultured fibroblasts.

### CTGF expression is regulated by MAS-specific ligands

**Fig 2:**
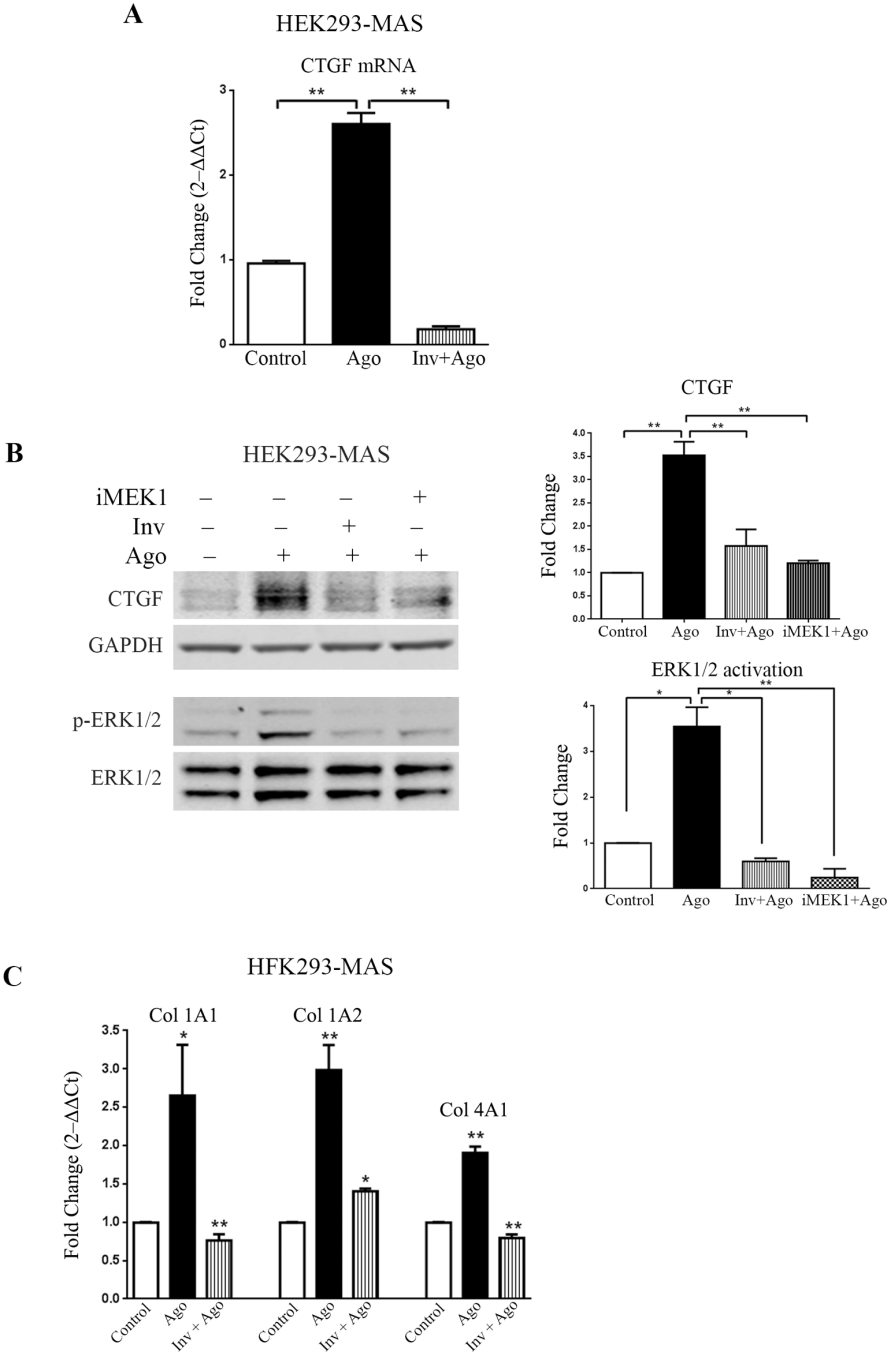
Agonist activated MAS receptor induces CTGF through ERK1/2 and regulates collagen expression in HEK293 cells stably expressing MAS. **(A)** Real-time PCR analysis shows significant upregulation of CTGF expression in response to MAS receptor agonist (AR234960; 10μM); while MAS inverse-agonist (AR244555; 10μM) along with agonist suppresses the expression of CTGF below the basal level. **(B)** Western-blot showing significant upregulation of CTGF in MAS agonist (AR234960) activated samples whereas CTGF expression decreases in presence of inverse-agonist (AR244555). MAS activated by AR234960 induces phosphorylation of ERK1/2, MAS inhibition by the inverse-agonist (AR244555) reduces ERK1/2 activation. MAS expressing HEK293 cells also show significant down-regulation of CTGF in presence of MEK1 inhibitor (PD98059). CTGF and p-ERK1/2 expression were normalized by GAPDH and ERK1/2 respectively. The western blot image shown is a representative of all the experiments done under similar experimental conditions and data from multiple experiments quantitated and cumulative data were presented as bar graph, (*p<0.05; **p<0.01). **(C)** Bar graphs showing real-time PCR analysis of different collagen sub-types (Col1A1, Col1A2 and Col4A1) [represented as fold increase (2^‒ΔΔCt^)] in HEK293-MAS cell line. Activated MAS induces collagen synthesis while repression of MAS receptor by its inverse agonist (AR244555) shows significant down-regulation of the same collagen sub-types (*p<0.05; **p<0.01). RT-qPCR was normalized by GAPDH.

To examine the relation between CTGF expression and ligand-modulation of MAS, we used the doxycycline inducible MAS expressing HEK293-MAS cell line. HEK293-MAS express MAS receptor only when treated with doxycycline as described in our previous studies [18]. We treated induced cells with the non-peptide MAS-agonist, AR234960 (Ago, 10μM) and the inverse-agonist AR244555 (10μM) along with the agonist (Inv+Ago). Treatment of MAS agonist increased both CTGF mRNA (>2.5 fold) and protein (>3 fold) levels compared to induced cells not treated with agonist. CTGF expression was significantly reduced by MAS inverse-agonist treatment, demonstrating specificity of CTGF expression in MAS-agonist activated cells (Fig 2A and 2B). MAS induced expression level of CTGF in HEK293-MAS cells (3.52±0.29) is comparable to the expression in cardiac tissue (3.59±0.43), which showed almost similar relative fold increase of CTGF in either diseased condition or in MAS-agonist (AR234960) treated induced cells compared to their respective control. We examined physiological peptide ligands of MAS such as Ang(1–7) (10μM; Sigma, MO) and NPFF (10μM; CCF-LRI, OH). Ang(1–7) did not elicit CTGF expression compared to untreated control (S2 Fig). The neuropeptide NPFF elicited CTGF expression (S2 Fig), which could also be blocked by the inverse agonist AR244555 (Data not shown). However, the magnitude of CTGF response was significantly smaller than that elicited by AR234960. Therefore, we have used the non-peptide agonist AR234960 in rest of the study.

**Fig 3:**
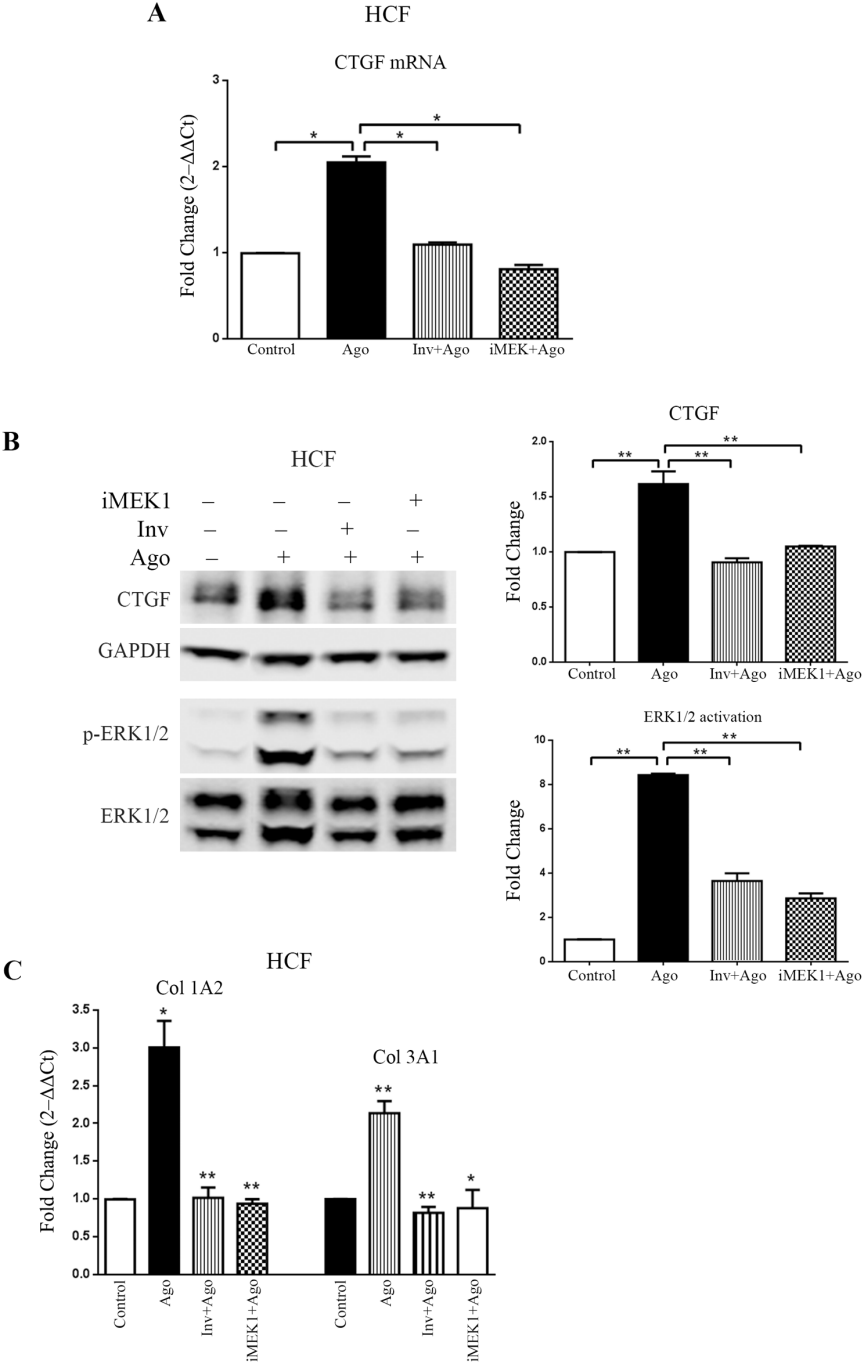
MAS receptor present on adult human cardiac fibroblast primary cells induces CTGF and collagen expression in response to its agonist. **(A)** Real-time PCR analysis shows significant upregulation of CTGF expression in response to MAS agonist (AR234960; 10μM); while MAS inverse-agonist (AR244555; 10μM) along with agonist (AR234960; 10μM) reduced CTGF expression significantly. CTGF expression decreases when MAS signaling is blocked by MEK1 inhibitor treatment. **(B)** Western-blot showing significant upregulation of CTGF in HCF cells treated with MAS agonist (AR234960; 10μM); the CTGF expression decreases when treated with inverse-agonist (AR244555; 10μM). MAS agonist (AR234960) activation also induces phosphorylation of ERK1/2 in HCF cells. In MAS agonist (AR234960) treated HCF cells, CTGF expression as well as ERK1/2 activation were significantly down-regulated in presence of MEK1 inhibitor (PD98059). CTGF expression and p-ERK1/2 levels were normalized by GAPDH and total ERK1/2 respectively. The western blot image shown is a representative of all the experiments done under similar experimental conditions and data from multiple experiments quantitated and cumulative data were presented as bar graphs. **(C)** Bar graphs showing real-time PCR analysis of different collagen sub-types (Col1A2 and Col3A1) [represented as fold increase (2^‒ΔΔCt^)] in HCF cells. Activated MAS induces collagen synthesis while inhibition of MAS receptor shows significant down-regulation of the same collagen sub-types. Inhibiting MEK1 also reduces expression of the same collagen sub-types. RT-qPCR was normalized by GAPDH. (*p<0.05; **p<0.01).

The expression of CTGF induced by the MAS agonist AR234960 in HEK293-MAS cells led us to examine whether MAS agonist activation leads to CTGF expression in adult primary human cardiac fibroblast (HCF) cells in culture. We confirmed expression of MAS in HCF primary cells by measuring the level of mRNA by RT-qPCR (S4 Fig). Upon treatment with MAS non-peptide Ago the expression of both mRNA and protein levels of CTGF was significantly increased in HCF cells (Fig 3A and 3B). The MAS inverse-agonist down regulated the expression of CTGF. Thus, these results indicate that MAS receptor activation can induce CTGF expression in cells.

### MAS induced ERK1/2 phosphorylation is essential for CTGF expression

We have previously shown that the agonist AR234960 activated MAS signals by means of G protein activation [18]. To identify the specific signaling pathway downstream of the G protein that is involved in CTGF expression, we studied activation of kinases, ERK1/2, p38MAPK, JNK, JAK2, PKC-γ and the transcription modulator STAT3 in HEK293-MAS cells as well as in HCF cells (Data not shown). ERK1/2 was significantly phosphorylated during agonist AR234960 treatment. When ERK1/2 activation pathway was blocked by inhibiting MEK1, the MAS agonist AR234960 activated ERK1/2 phosphorylation was reduced (>3 fold) in HEK293-MAS cells. iMEK1 lowered the basal phosho-ERK1/2 level in HEK293-MAS cell line (Fig 2B). The iMEK1 treatment also down-regulated MAS agonist AR234960 activated CTGF protein in both HEK293-MAS cell line (~1.5 fold) and HCF cells (>2.5 fold) (Figs 2B and 3B). CTGF mRNA level was also significantly decreased by iMEK1 treatment (Fig 3A). Thus MAS-phospho-ERK1/2 signaling directly mediates regulation of CTGF. We also observed phosphorylation of JAK2, STAT3 and p38MAPK after MAS agonist treatment of cells. However, pharmacological blockade of these molecules did not affect the agonist AR234960-induced expression of CTGF (S4 Fig).

### MAS signaling through ERK1/2 regulates collagen gene expression

As shown in Fig 1E, increased expression of collagen gene sub-types Col1A1, Col1A2, Col3A1 and Col4A2 is correlated with increased expression of MAS in HF samples. In the agonist AR234960 treated HEK293-MAS cells, we found marked increase of Col1A1, Col1A2 and Col4A1 compared to control cells not treated with the agonist (Fig 2C). This increase was inhibited by the inverse-agonist AR244555. Expression of other collagen sub-types was not altered (S5 Fig). In HCF cells collagen 1A2 and Collagen 3A1 were significantly increased upon MAS agonist AR234960 treatment (Fig 3C). This increment was reduced to basal level in the presence of MAS inverse-agonist AR244555 compared to agonist treated condition (Fig 3C). Collagen sub-types 1A1, 2A1, 4A1 and 4A2 were examined, but there was no significant difference compared to control HCF (S5 Fig). In both cell types, agonist AR234960 treatment combined with iMEK1 inhibited collagen sub-type gene expression that is characteristic for the cell, implying that MAS signaling through ERK1/2 regulates collagen gene expression.

### Agonist AR234960-induced CTGF regulates collagen expression

**Fig 4:**
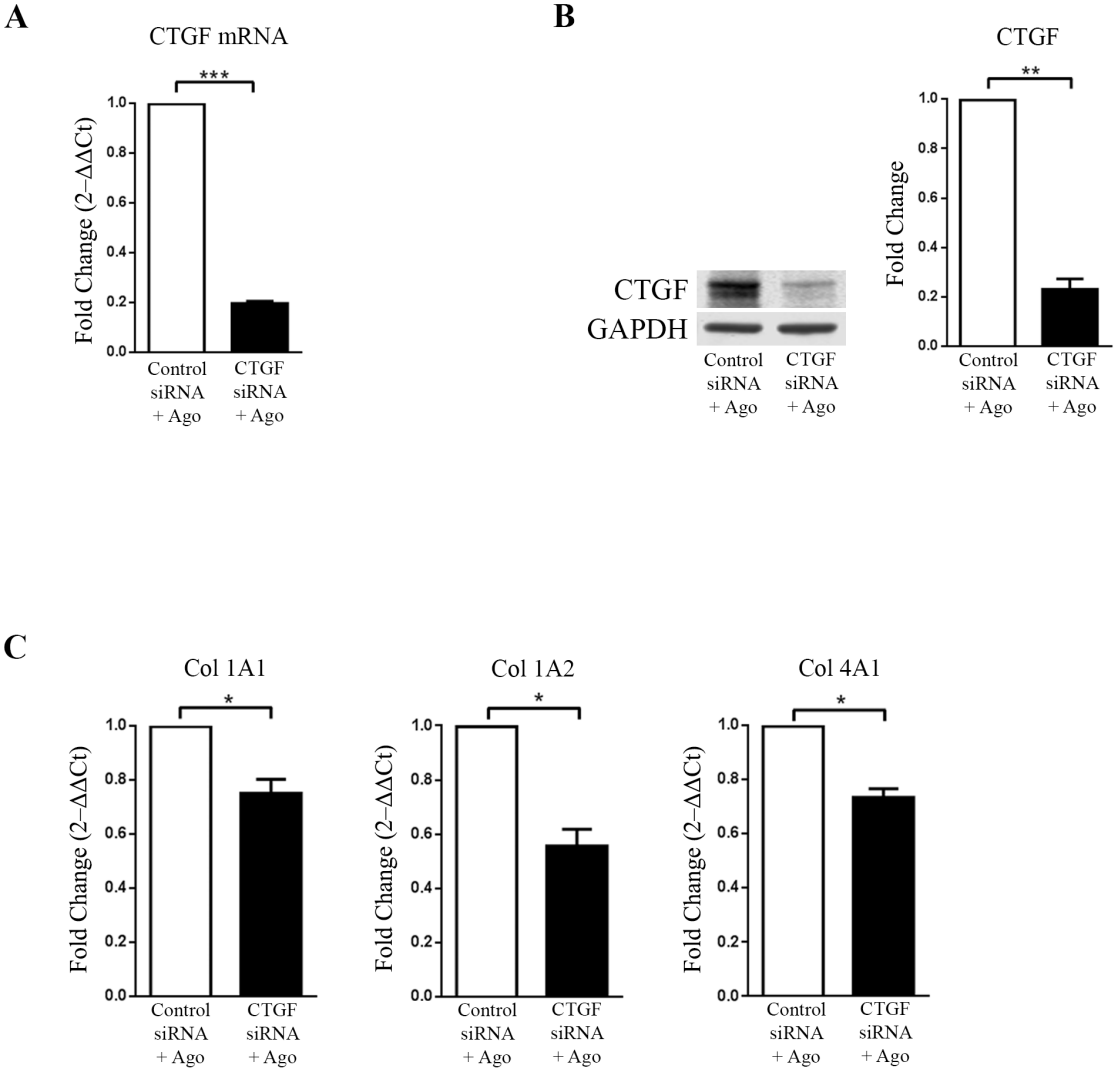
CTGF regulates collagen in HEK293-MAS cells treated with MAS agonist (AR234960). MAS expressing HEK293 cells were transiently transfected with control siRNA and CTGF siRNA followed by treatment with MAS agonist (AR234960). (A) Real-time PCR analysis confirms CTGF down-regulation by CTGF siRNA as compared to control siRNA transfected and followed by MAS agonist treatment. GAPDH was used as loading control. CTGF siRNA is specific and has no off-target effect on MAS expression. (B). Western-blot confirmation of CTGF protein levels. The western blot image shown is a representative of all the experiment done under similar experimental condition and data from multiple experiments quantitated and cumulative data were presented as bar graphs (**p<0.01) (C) Same set of sample was used for RT-qPCR analysis of collagen sub-types. Col 1A1, Col 1A2 and Col 4A1 were significantly down-regulated in MAS induced CTGF siRNA transfected samples compared to MAS induced control siRNA samples. RT-qPCR and western blot were normalized by GAPDH. (*p<0.05; **p<0.01; ***p<0.001).

**Fig 5:**
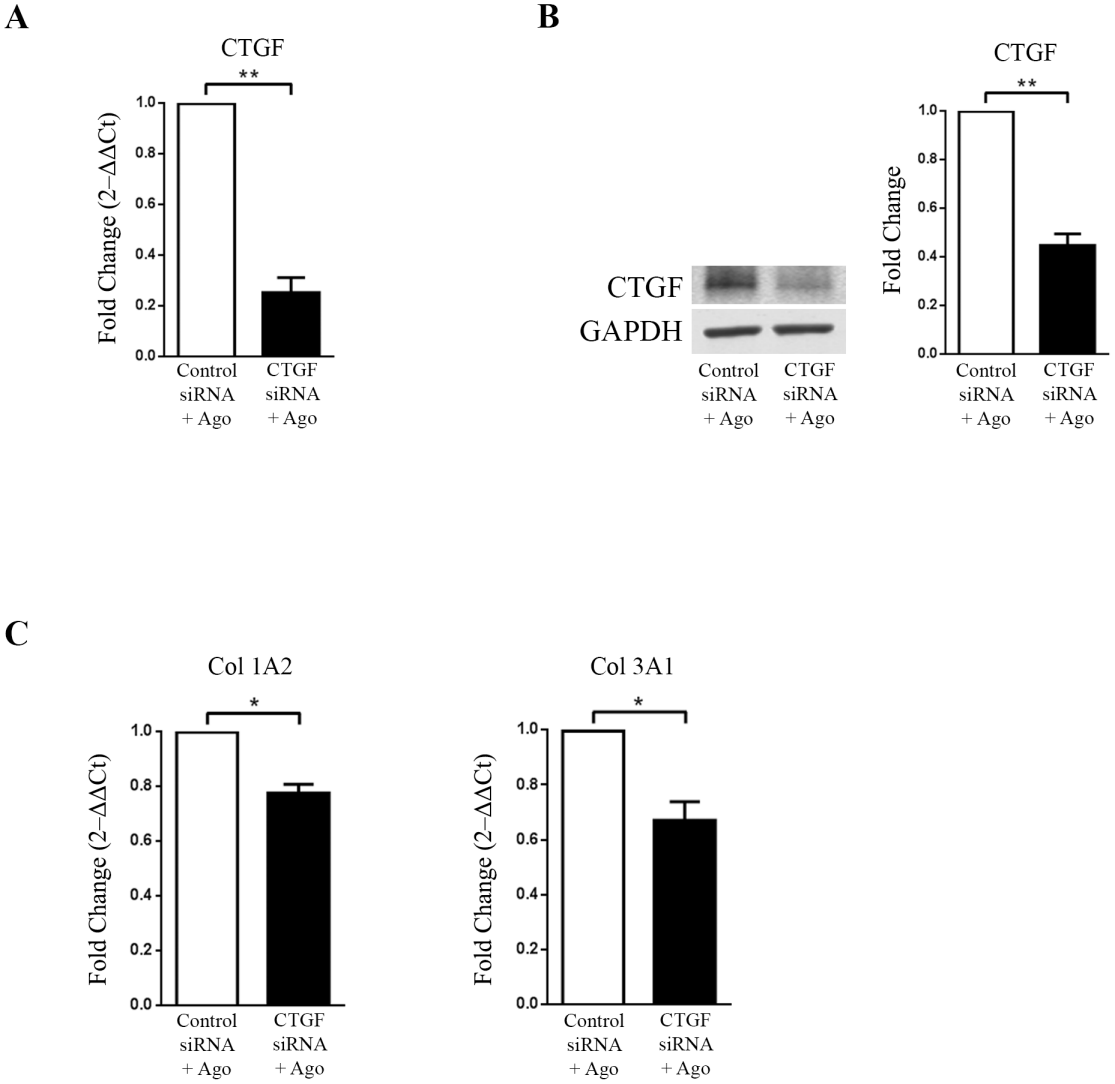
CTGF regulates agonist (AR234960) induced collagen expression in HCF cells. HCF cells were transiently transfected with control siRNA and CTGF siRNA followed by treatment with MAS agonist (AR234960). **(A)** Real-time PCR analysis confirms CTGF down-regulation by CTGF siRNA as compared to control siRNA transfected and followed by MAS agonist treatment. GAPDH was used as loading control. CTGF siRNA did not affect MAS expression in HCF (S3 Fig). **(B)**. Western-blot confirmation of CTGF protein levels. The western blot image shown is a representative of all the experiments done under similar experimental condition and data from multiple experiments quantitated and cumulative data were presented as bar graphs (left) (**p<0.01) **(C)** Same set of sample was used for RT-qPCR analysis of collagen sub-types. Col1A2 and Col3A1 were significantly down-regulated in MAS induced CTGF siRNA transfected samples compared to MAS induced control siRNA samples. RT-qPCR and western blot were normalized by GAPDH. (*p<0.05; **p<0.01).

CTGF expression is reported to boost extracellular matrix protein expression by cardiac fibroblasts [7]. Therefore we examined whether MAS induced CTGF in cells directly regulates collagen gene expression. We used siRNA mediated knock-down of CTGF and then examined collagen gene expression in both HEK293-MAS and HCF cells. After transient transfection with control and CTGF siRNA, doxycycline induced HEK293-MAS cells were treated with MAS agonist AR234960. HCF cells were transiently transfected with the control and CTGF siRNA, followed by treatment with MAS agonist AR234960. CTGF expression measured by RT-qPCR and western blot was significantly down-regulated (~5 fold) by CTGF siRNA in MAS expressing HEK293 cells as well as in HCF cells, thus confirming its effective delivery and action within cells (Figs 4A, 4B, 5A and 5B).

HEK293-MAS cells transfected with CTGF siRNA, followed by MAS agonist treatment showed significant down-regulation of Col1A1, Col1A2 and Col4A1 compared to cells transfected with control siRNA and MAS agonist treatment (Fig 4C). In HCF cells, CTGF siRNA and MAS agonist treatment led to significant down regulation of CTGF along with reduction of collagen type 1A2 and Col3A1 expression (Fig 5C). These data confirm that up-regulation of CTGF expression by the agonist AR234960 activated MAS is essential for collagen expression in HCF cells. This finding is relevant for proposing that MAS-ERK1/2-CTGF pathway might be an important regulator of ECM remodeling associated with HF progression (see Discussion).

## Discussion

In the present study, we observed significant correlation between expression of MAS and CTGF in human heart tissue. Presence of MAS transcripts and protein in all chambers of human hearts, including the coronary arteries was previously shown [14–16]. Cardiac MAS expression profile was shown to change upon different pathological stimuli, implying that MAS may participate in the establishment and/or progression of HF [28]. We analyzed HF samples with severely compromised EF as the common feature but with diverse clinical diagnoses, as presented in Table 1. We found that MAS expression is increased in the HF group compared to the NF group and the increase of MAS was associated with increased CTGF expression (Fig 1A, 1B and 1C). The increase of MAS in HF and the correlation between MAS and CTGF are not affected by patient characteristics including age, gender, race and medications in this analysis. Cardiac remodeling was evident in the HF samples based on staining for CTGF and collagen which are indicative of hypertrophy and fibrosis (Fig 1D). Accordingly, collagen gene expression was also increased in HF compared to NF (Fig 1E). CTGF is suggested as a potential biomarker of HF [24]. CTGF is also a factor associated with fibrosis of organs including lung, kidney, skin, and heart [7,8,12,25]. These observations prompted us to investigate CTGF as a mediator of cardiac fibrosis linked to MAS signaling.

MAS is a constitutively active GPCR; hence, changes in expression levels of MAS could directly alter signaling pathways without requiring a ligand. In addition, various endogenous agonists reported to bind to MAS including the neuropeptide FF (NPFF), Ang1–7 and alamandine could produce complex signals. Studies in animal models have shown that Ang1–7 is a key player in the regulation of sympathetic outflow in HF [29, 30] and cellular hypertrophy and myofibroblast transformation [31]. Pharmacological blockade of MAS has been shown to attenuate cardiac hypertrophy during pregnancy [32] and increase collagen and fibronectin levels [33]. Thus MAS could regulate cardiac hypertrophy and expression of fibrotic marker proteins. Hence it is important to understand complexities underlying the activation of MAS-CTGF-collagen signaling.

To understand the mechanistic relationship between MAS, CTGF and collagen, we examined whether specific agonist activation of MAS in cells induces expression of CTGF and collagen genes. Previous studies have shown that AR234960 and AR244555 modulate G protein signaling by MAS in a dose dependent manner [16, 18] in MAS overexpressing HEK293 cells. The MAS agonist AR234960 treatment increased phosphorylation of ERK1/2, expression of CTGF protein (~3.5 fold) and transcription of collagen genes as shown in Fig 2. Blocking MAS activation by the inverse-agonist AR244555 treatment inhibited the responses in MAS overexpressing HEK293 cells. We next examined whether MAS agonist AR234960 treatment of HCF primary cells expressing endogenous levels of MAS activates CTGF expression. The MAS agonist treatment increased phosphorylation of ERK1/2 >8 fold and CTGF protein expression >1.5 fold. Furthermore, transcript levels for distinct collagen genes were up-regulated. The collagen gene subtype expression responses were consistently reproduced in a cell-type specific manner in all experiments. The delta-deltaCt (and deduced 2^-ΔΔCt^) values for unaffected collagen subtype gene expression in HEK-MAS and HCF cells were not pursued because of no change, a redundant and negative result. All three responses were inhibited to basal level when the agonist AR234960 activated HCF cells were treated with MAS inverse-agonist AR244555. It is evident that inactive MAS receptor leads to inhibition of CTGF and collagen expression in both cell types. These data establish that CTGF and collagen are MAS regulated genes. MAS agonist treatment potentiates MAS transcript level in HCF (S3 Fig). Thus, these non-peptide ligands are useful pharmacological tools to examine MAS function.

To map the signaling events that regulate CTGF and collagen expression mediated by the MAS agonist AR234960, we surveyed activation of several protein kinases and found that ERK1/2 activation was concurrent with expression of CTGF and collagen. Although, AR234960 treatment induced the activation of JAK2/STAT3 and p38 MAPK in HEK-MAS cells (S4 Fig); inhibiting these pathways did not interfere with CTGF expression. However, when the effect of inhibition of ERK1/2 phosphorylation was evaluated, we observed that inhibitor of MEK1 (iMEK1; PD98059) blocked the activation of ERK1/2, which in turn inhibited the expression of CTGF as well as collagen in HEK293-MAS cells and in HCF primary cells. To evaluate whether collagen expression is mediated by MAS-induced CTGF, we used RNAi based knockdown of CTGF expression in MAS agonist AR234960 treated cells. In both HEK293-MAS and HCF cells, CTGF expression was concurrent and comparable with the collagen expression, demonstrating that CTGF acts as a specific regulator of collagen expression. Collagen gene sub-types are known to be regulated in tissue and cell specific manner by multiple factors and in this study we found CTGF indeed activated distinct collagen subtypes in HEK293-MAS and HCF cells. Thus MAS activation could contribute to extracellular matrix remodeling during HF.

The in vivo regulation of MAS-CTGF-collagen pathway is anticipated to depend on interaction of MAS with physiological ligands, neuropeptide FF (NPFF), Ang1–7, angiotensin fragments, angioprotectin, and alamandine that may be locally produced or delivered through circulation [4, 12]. As shown in S2 Fig, we did not see CTGF induction when induced HEK293-MAS cells were treated with Ang1-7. Furthermore, we did not observe CTGF expression response to the RAS peptide hormone Ang1-7 and other MAS agonists AVE0991 and CGEN 856S (Data not shown). In contrast, the neuropeptide hormone NPFF elicited a CTGF response that was weaker compared to the non-peptide MAS agonist AR234960. This finding is consistent with the observations that classical G protein signaling and desensitization response pathways are weakly activated when Ang1–7 and other angiotensin fragments bind to MAS. The NPFF and MBP7-bound MAS produced G protein signaling similar to AR234960, but differed in magnitude and desensitization of response [16, 18]. Taken together, these findings suggest that in vivo ligands for MAS such as Ang1-7 and NPFF may induce functional selectivity signaling. The non-peptide MAS agonist AR234960 appears to mimic the NPFF effects in activating the CTGF-collagen signaling which is efficiently blocked by the inverse-agonist AR244555 treatment. The compound AR244555 may be a promising pharmacological tool for preventing fibrogenic actions of MAS in vivo.

Few studies have shown that neonatal as well as adult cardiomyocytes contribute to extracellular matrix remodeling by regulating CTGF expression in response to either by certain growth factors or by stimulators. Cardiac fibroblasts, in connection with cardiomyocytes, aid in fibrosis development as fibroblasts are one of the important contributors of CTGF production not only in cardiac tissue, but also in other tissues. In our study, we have tried to dissect the role of cardiac fibroblast during MAS induction and pathological conditions of heart. Regulation of CTGF and collagen in HCF induced by native MAS shown here for the first time represents a model study in cardiac fibroblasts, which are major constituents influencing the functions of the heart. Chronic elevations in circulating hormones increase cardiac fibroblast proliferation and enhance collagen synthesis that accounts for myocardial fibrosis. Deposition of collagen prevents heart tissue from proper functioning by causing stiffness of cardiac tissue and lowering of cardiac output [2, 34]. The master signal for this may involve locally generated paracrine factor CTGF, which can cause imbalance of ECM ultimately leading to abnormal tissue function and organ failure. HCF studies also showed significant increase of collagen I and III which is observed in human HF [2,34–36]. The levels of CTGF in tissue correlate with the degree and severity of fibrosis [11,36] as well as its role as a hypertrophic factor for cardiomyocytes [37]. The plasma CTGF level is also associated with HF in dilated cardiomyopathy patients [38, 39]. Finding that cardiac fibroblasts produce CTGF and collagen downstream of MAS provides the basis for consideration of MAS blockade as a therapy for cardiac disease states, specifically to combat HF and fibrosis. Current interventions available to decrease CTGF expression and fibrosis include ACE inhibition and angiotensin receptor blockade [6].

In conclusion, this study demonstrates that MAS mediated CTGF production and collagen deposition may play a role during cardiac dysfunction. MAS receptor signals through phosphorylation of ERK1/2. For the first time, we demonstrate CTGF expression downstream of MAS and its significance in cardiac disease. CTGF, being an important regulator of ECM balance for proper heart functioning, would be a target for drug development. However, additional research is needed to fully dissect the role of CTGF on cardiac fibroblasts and HF. A thorough understanding of mechanism of MAS-CTGF signaling pathway in heart disease might provide additional HF treatment strategies.

## Supporting information

S1 Fig Tissue from compromised hearts were used in this study. Non-failing (NF) and Failing (HF) groups’ average age was 48.5±4.6 and 57.6±2.0 respectively and there was no significant age difference between these two groups (left). But there was a significant difference (p value<0.0001) in cardiac function, as percentage ejection fraction of NF was 61.0±2.11 and of HF was 21.2±2.55 (right).
(PDF)

S2 Fig Testing of MAS ligands Ang1-7, NPFF and small molecule ligand AR234960 (Ago) to check CTGF expression level in HEK293-MAS induced cell line. Western blot shows upregulation of CTGF in HEK293-MAS cells in response to MAS agonists such as AR234960 (Ago) and NPFF, but Ang1-7 could not elicit the level of CTGF in this experiment. Use of inverse-agonist (Inv) along with MAS agonist down-regulates the CTGF expression (data not shown) in combination with the agonists where applicable. GAPDH was used as loading control.
(PDF)

S3 Fig Confirmation of MAS expression in HCF primary cells. MAS mRNA expression was analyzed in HCF cells, by first preparing total cDNA by random priming followed by gene-specific PCR. Agarose gel shows the presence of MAS cDNA (160 bp long PCR products) and higher level of MAS transcript in response to MAS agonist (AR234960).
(PDF)

S4 Fig Inhibition of p38MAPK and JAK2 could not alter CTGF in HEK-MAS cells. HEK-MAS cells were induced and treated with Ago, Ago with p38MAPK inhibitor (SB203580, Cell Signaling Technology) and Ago with JAK inhibitor (420099; EMD, Millipore). Western blot showing phosphorylation of p38MAPK (Panel: top left), JAK2 and STAT3 (Panel: top right) in response to MAS Agonist (Ago). As expected, treatment of HEK-MAS cells with p38MAPK inhibitor and JAK inhibitor along with Ago leads to almost complete abolition of activation of either p38MAPK or JAK2 and STAT3 by blocking addition of phosphate group to it. The same samples were checked for CTGF expression (Panel: bottom), MAS Ago induced CTGF expression in samples treated either alone with Ago or in combination with inhibitors (p38MAPK Inhibitor and JAK inhibitor). GAPDH was used as loading control.
(PDF)

S5 Fig Collagen sub-types remained non-responsive to MAS agonist (AR234960). Expression level of collagen subtypes such as Col2A1, Col3A1 and Col4A2 were analyzed by real-time PCR and changes were found to be non-significant in response to MAS agonist in HEK-MAS293 cells. In HCF, expression of Col1A1, Col2A1, Col4A1 and Col4A2 did not change in AR234960 treated cells compared to control. RT-qPCR was normalized by GAPDH. (NS = Not Significant).
(PDF)
