# Supplementary figures and images for: Connective tissue growth factor dependent collagen gene expression induced by MAS agonist AR234960 in human cardiac fibroblasts
Source: PLoS One. 2017 Dec 29;12(12):e0190217. doi: 10.1371/journal.pone.0190217 (PMC5747466; doi:10.1371/journal.pone.0190217)

**S1 Fig**

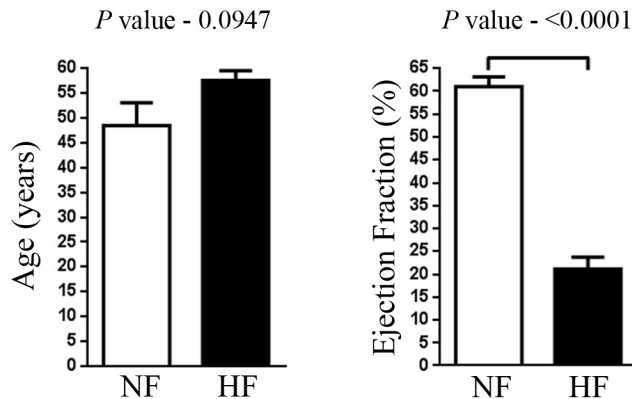

Supplement: S1 Fig — Non-failing (NF) and Failing (HF) groups’ average age was 48.5±4.6 and 57.6±2.0 respectively and there was no significant age difference between these two groups (left). But there was a significant difference (p value<0.0001) in cardiac function, as percentage ejection fraction of NF was 61.0±2.11 and of HF was 21.2±2.55 (right). (PDF) [file pone.0190217.s001.pdf]

**S2 Fig**

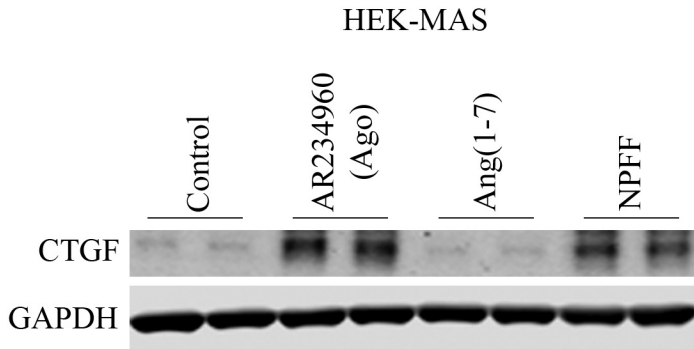

Supplement: S2 Fig — Western blot shows upregulation of CTGF in HEK293-MAS cells in response to MAS agonists such as AR234960 (Ago) and NPFF, but Ang1-7 could not elicit the level of CTGF in this experiment. Use of inverse-agonist (Inv) along with MAS agonist down-regulates the CTGF expression (data not shown) in combination with the agonists where applicable. GAPDH was used as loading control. (PDF) [file pone.0190217.s002.pdf]

### S3 Fig

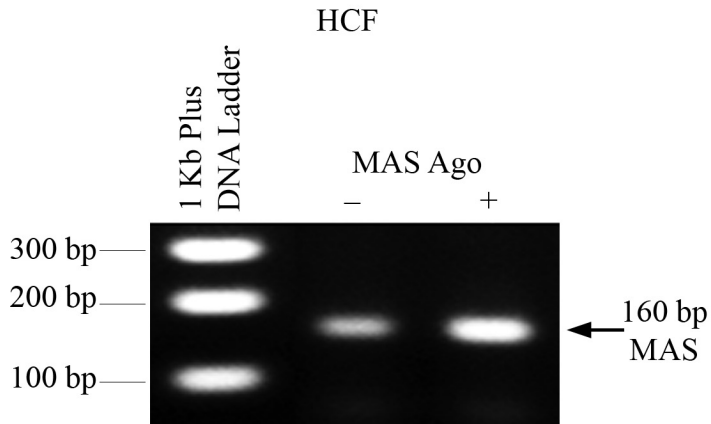

Supplement: S3 Fig — MAS mRNA expression was analyzed in HCF cells, by first preparing total cDNA by random priming followed by gene-specific PCR. Agarose gel shows the presence of MAS cDNA (160 bp long PCR products) and higher level of MAS transcript in response to MAS agonist (AR234960). (PDF) [file pone.0190217.s003.pdf]

**S4 Fig**

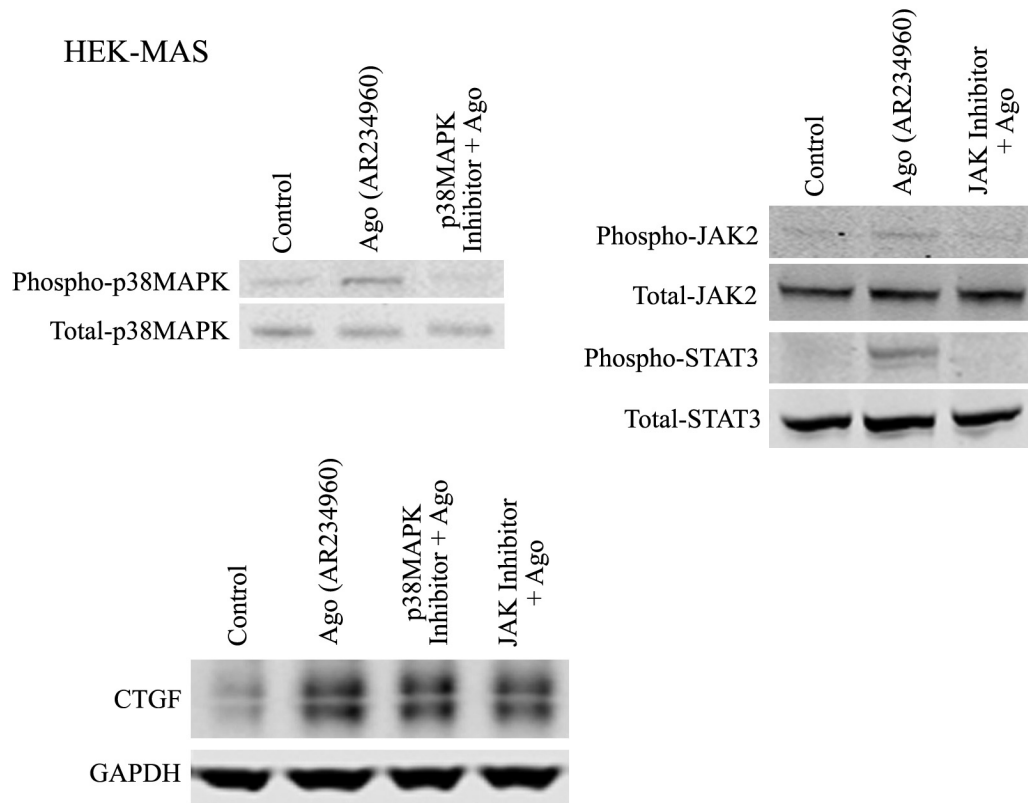

Supplement: S4 Fig — HEK-MAS cells were induced and treated with Ago, Ago with p38MAPK inhibitor (SB203580, Cell Signaling Technology) and Ago with JAK inhibitor (420099; EMD, Millipore). Western blot showing phosphorylation of p38MAPK (Panel: top left), JAK2 and STAT3 (Panel: top right) in response to MAS Agonist (Ago). As expected, treatment of HEK-MAS cells with p38MAPK inhibitor and JAK inhibitor along with Ago leads to almost complete abolition of activation of either p38MAPK or JAK2 and STAT3 by blocking addition of phosphate group to it. The same samples were checked for CTGF expression (Panel: bottom), MAS Ago induced CTGF expression in samples treated either alone with Ago or in combination with inhibitors (p38MAPK Inhibitor and JAK inhibitor). GAPDH was used as loading control. (PDF) [file pone.0190217.s004.pdf]

**S5 Fig**

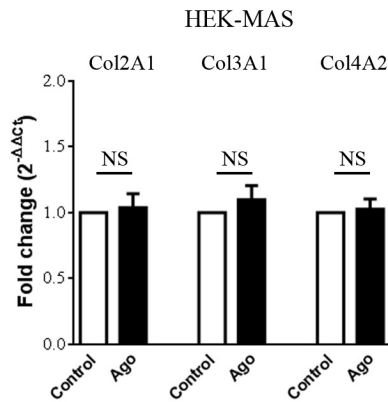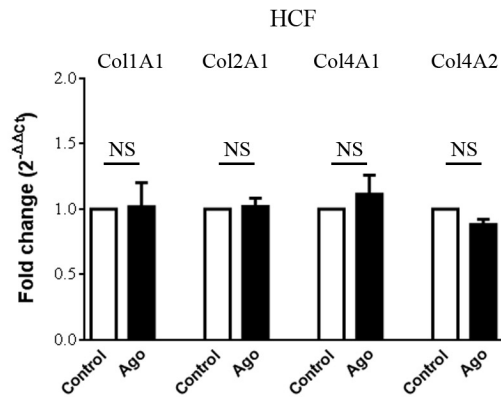

Supplement: S5 Fig — Expression level of collagen subtypes such as Col2A1, Col3A1 and Col4A2 were analyzed by real-time PCR and changes were found to be non-significant in response to MAS agonist in HEK-MAS293 cells. In HCF, expression of Col1A1, Col2A1, Col4A1 and Col4A2 did not change in AR234960 treated cells compared to control. RT-qPCR was normalized by GAPDH. (NS = Not Significant). (PDF) [file pone.0190217.s005.pdf]
